# Processing-Induced Modifications of Camel Milk Immunoglobulins and Lactoferrin: Implications for Immunocompromised Pediatric Populations and Therapeutic Applications

**DOI:** 10.3390/foods15061028

**Published:** 2026-03-16

**Authors:** Omar A. Alhaj, Mohammed O. Ibrahim, Nour A. Elsahoryi, Ola D. Al-Maseimi

**Affiliations:** 1 Department of Nutrition, Faculty of Pharmacy and Medical Sciences, University of Petra, Amman 11196, Jordan; nour.elsahoryi@uop.edu.jo; 2 Department of Nutrition and Food Technology, Faculty of Agriculture, Mutah University, Karak 61710, Jordan; mohammedomar@mutah.edu.jo; 3 Department of Nutrition and Food Science, Faculty of Allied Medical Sciences, Al-Balqa Applied University, Al-Salt 19117, Jordan; olaalmaseimi@bau.edu.jo

**Keywords:** camel milk, immunoglobulins, lactoferrin, thermal processing, non-thermal processing, immunocompromised children, pediatric immunodeficiency

## Abstract

Immunocompromised pediatric populations (children with inborn errors of immunity, HIV infection, and cancer, as well as those undergoing hematopoietic stem-cell transplantation) have severe nutritional challenges, with malnutrition depending on the underlying condition. Camel milk (CM) represents a culturally accessible, high-quality nutritional parameter and functional food naturally enriched with particular immunological components such as heavy-chain antibodies that represent 75% of total immunoglobulins (IGs) and lactoferrin (LF) at a concentration 3–5 times higher than bovine milk (BM). However, there is a critical processing paradox: the thermal treatments required for the microbiological safety of immunosuppressed children who show a 20-fold greater susceptibility to foodborne pathogens degrade the therapeutic bioactive proteins. This comprehensive review provides a systematic evaluation of processing-induced modifications of CM IGs and LF, which involve thermal and non-thermal technologies, and their effects on the molecular structure and biological function. Emerging alternatives such as high-pressure processing (HPP), pulsed electric fields, and strategic fermentation show promising bioactivity retention without compromising safety. Critical knowledge gaps remain in the structure–function relationships of processed CM proteins, necessitating evidence-based optimization strategies to balance microbiological safety with clinically relevant immunomodulatory functions for vulnerable pediatric populations.

## 1. Introduction

**Figure 1:**
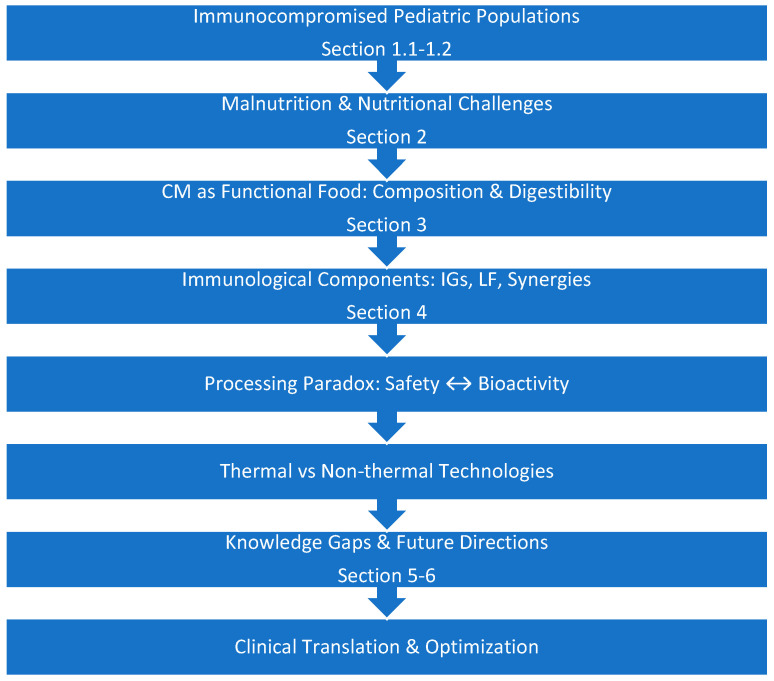
Review structure and conceptual flow.

The structure of this review aims to address: (1) the global issue of immunocompromised children burden of disease and nutrition (Section 1.1 and Section 1.2), (2) the compositional and immunological benefits of CM (Section 2 and Section 3), (3) processing-induced changes and gaps in knowledge (Section 4), and (4) the opportunities to optimize therapeutic uses (Section 5), as shown in the flow diagram in Figure 1.

### 1.1. Global Burden of Pediatric Immunodeficiency

Pediatric competence is a core determinant of survival, with disruption resulting in recurrent severe infection, immune dysregulation, and malignancy [1,2,3]. Inborn errors of immunity (IEI) now encompass several hundred distinct monogenic entities, with the 2019 IUIS classification identifying 430 distinct IEI phenotypes contributing to approximately 404 clinically recognizable PIDD presentations [4,5]. A 14-year analysis of the US PHIS database encompassing 17,234 pediatric patients documented an incidence of new PIDD diagnoses of 2.8 per 1000 hospital discharges and an overall hospitalization rate of 6.3 per 1000 discharges between 2004 and 2018, with an in-hospital mortality of 4.85% [2].

The apparent rarity of pediatric IEI is driven by under-recognition rather than true low prevalence [6]. Out of 81 children evaluated at the Children’s Hospital of the Alexandria University, 61.7% met the WHO criteria of confirmed PIDD, with an antibody deficiency predominance of 34%, well-defined syndromes of IEI accounting for 30%, combined immune-deficiencies accounting for 16%, phagocytic defects accounting for 14%, immune dysregulation disorders accounting for 4%, and complement deficiencies accounting for 2% [3]. The mean age of diagnosis was 27.4 months, and 28.4% succumbed to the complications associated with infection, with the leading factor being the delay in diagnosis [3]. A French pediatric intensive care unit study found confirmed primary immunodeficiency diseases in 6.5% of children hospitalized for severe infection, with 87% presenting immunological anomalies, including low natural killer lymphocyte levels (41%) and abnormal B-cell distribution (25%) [7].

### 1.2. Nutritional Challenges in Immunocompromised Pediatric Populations

Dysfunction of the immune system and malnutrition are mutually reinforcing in children in such a way that infection, metabolic stress, and growth failure develop in a self-perpetuating cycle [8,9,10]. Severe malnutrition with protein-energy deficiency and micronutrient deficiency undermines the integrity of epithelial barriers; impairs secretory IgA in mucosal surfaces; blunts complement activity, neutrophil chemotaxis, and oxidative burst; and induces thymic atrophy and lymphopenia and T-cell-dependent antibody responses [8,9]. Defective host defenses also increase recurrent and severe infections—especially diarrhea and pneumonia—that further slow down appetite, elevate resting energy expenditure, and lead to the loss of nutrients, further propagating the malnutrition–infection cycle [11].

Nutritional stress is superimposed on direct viral immune injury and antiretroviral therapy (ART) toxicities in children with HIV [12,13]. An East African cohort meta-analysis showed a prevalence of underweight children with 41.6% wasting and 24.65% stunting among HIV-infected children [14], which were significantly higher than the prevalence of anthropometric deficits in HIV-uninfected children, and these anthropometric deficits were strongly correlated with an increased risk of infection-related morbidity and death before 2 years of age [14,15]. Another category of immunocompromised patients with nutritional risk is children with cancer; both chemotherapy, radiotherapy, and corticosteroids are known to cause nausea, vomiting, mucositis, diarrhea, and increase lean body mass catabolism [16,17]. These are the contemporary reports of malnutrition at 6–51%, with under-nutrition also being a consistent predictor of increased infectious complications, dose-limiting chemotherapy toxicities, delays in treatment, and reduced overall survival [16,18,19,20].

Severe combined immunodeficiency (SCID) is a syndrome that occurs at the far end of the spectrum of nutritional variability, where 54–88% of patients with SCID fail to thrive at the time of diagnosis [21]. SCID studies of indirect calorimetry show hypermetabolism in about 93% of the SCID patients, with a resting energy expenditure (REE) often over 150% of the predicted values, suggesting that energy deficits are underestimated and that both immune recovery and catch-up growth require higher than normal dietary prescriptions [21]. Children who have antibody deficiency and other types of IEI are generally reported as deficient in fat-soluble vitamins (A, D, E, and K) and trace elements (zinc, selenium, copper, and iron) [21,22]. In children with IEI undergoing hematopoietic stem-cell transplantation (HSCT) as the sole curative treatment of the most severe forms of the disease, nutritional issues become very severe [23]. Among 27 children with IEI who received HSCT, with 31 total transplant procedures, baseline nutritional deficits were reported for one-third of the patients: 33% had weight and/or height ≤ −2 standard deviations at baseline assessment, meeting criteria for severe malnutrition [23]. Forty percent of this group needed some type of nutritional support before transplantation, with 33% of them having enteral tube nutrition and 7% need long-term parenteral nutrition [23]. Nutritional dependency increased significantly after transplantation, 82% of these children needed parenteral nutrition with an average of 67 days, which indicates the severe mucosal damage, gastrointestinal intolerance, and inability to sustain oral intake, which is a typical feature of the initial post-HSCT period [23,24,25,26]. It is only after HSCT that full oral intake was re-established, with a mean of 154 days and a 5-month window, with totally intensive and technology-driven support being the only way of ensuring adequate delivery of macro- and micronutrients [23]. Although conventional protocols for micronutrient supplementation have been recorded, pre- and post-transplantation deficiencies in zinc, vitamin A, vitamin D, selenium, and copper were reported, and low levels of vitamin D at transplantation tapped vitamin D insufficiently only at transplantation, indicating that micronutrient restoration could have a direct effect on immune recovery outcomes [23].

However, there is pediatric evidence that is heterogeneous, with the majority of studies conducted on preterm neonates lacking primary immunodeficiency [21]. Trials of bovine LF in very-low-birth-weight preterm infants have shown mixed results, with some single-center studies reporting possible reductions in late-onset sepsis and necrotizing enterocolitis [27], but larger multicenter trials and updated systematic reviews reporting neutral or inconsistent effects on composite clinical outcomes [28,29,30]. Importantly, preterm babies without known primary immunodeficiency, perinatally acquired HIV, or active malignancy were used in virtually all LF and immunonutrition studies, so that, practically, there are no available translational data on immunonutrition of children with IEI, pediatric HIV, or cancer in the literature [27].

Systematic reviews find that the overall safety, nutrition, and potential benefits of raw CM are safe and nutritionally dense, and in particular, the benefits of the technology are evidence-based and may be useful in certain diseases, including autism spectrum disorder, milk allergy, and chronic infectious diarrhea [31,32,33,34,35]. Though most accessible studies are small, heterogeneous in terms of methods, and have no unified definition of outcomes [31,32]. All of these properties of milk allow CM to be a candidate food-based immunonutrition in immunocompromised children living in areas with camel livestock: local availability, cultural acceptance, and natural enrichment of CM with LF and IGs [31,32,36,37].

### 1.3. Study Objectives and Innovative Approaches

The objectives of this review are threefold: First, to comprehensively evaluate processing-induced modifications of CM IGs and LF, examining how thermal and non-thermal technologies affect molecular structure and biological function, with emphasis on structure-function relationships determining therapeutic efficacy. Second, to synthesize evidence regarding the clinical potential of processed CM immunoproteins for immunocompromised pediatric populations, examining mechanisms of action, existing clinical data, and knowledge gaps linking processing choices to therapeutic outcomes. Third, to provide an evidence-based framework for optimizing processing strategies that balance microbiological safety with bioactivity preservation, specifically tailored for therapeutic applications in vulnerable pediatric populations. These objectives are systematically addressed through: processing modifications evaluation (Section 4.1 and Section 4.2, clinical potential synthesis (Section 1.1, Section 1.2, Section 3.3 and Section 5.1), and optimization framework development (Section 4.4, Section 4.5, Section 5.2 and Section 6).

This review makes unique contributions by integrating: (1) molecular-level processing science with clinical pediatric immunology, a gap in current CM reviews that address composition and/or processing separately; (2) systematic assessment of structure–function relationships between processing-induced modifications and therapeutic bioactivity; and (3) evidence-based framework specifically adapted for immunocompromised pediatric populations in camel-producing regions, a critical processing paradox that does not exist in current literature.

## 2. Camel Milk as a Functional Food

### 2.1. Historical and Traditional Use in Pediatric Nutrition

CM has been an important nutritional resource in human history, especially for pastoralist people living in arid and semi-arid areas of Africa, the Middle East, and Central Asia, where CM has been used as a source of nutrition as well as a resource that operates within cultural, medicinal, and socioeconomic spheres [37,38]. The history of CM’s importance goes beyond being a source of sustenance, as it was commonly used as a source of much-needed nutrients in pre-industrial living environments during droughts and shortages of food, which helped to prolong the life of nomadic cultures in regions where other farm animals would not survive [36,39]. Traditional community-based therapies have been documented in the past; the medicinal value of CM, traditionally used as an indigenous therapy for jaundice, malaria, and digestive diseases [40]. Moreover, the cultural significance of CM is also reflected in its consumption in the preparation of traditional fermented products such as Shubat in Kazakhstan, Gariss in Sudan, and Susac in Kenya [41]. Within the ambit of pediatric nutrition in particular, CM has been historically recognized in traditional medicine systems, with classical medical texts from Middle Eastern, Asian, and African cultures describing the use of CM for children, often viewing it as the closest characteristics to the human mother’s milk based on its unique compositional profile [31,37]. The notion that CM resembles human milk is not only anecdotal but is backed by its peculiar composition, such as low lactose and cholesterol contents and high concentration of micronutrients like sodium, potassium, iron, copper, zinc, magnesium, and vitamin C [31]. Currently, the leading CM-producing countries are Kenya, Somalia, and Mali, making an annual amount of 1.16 million metric tons, 1.14 million metric tons, and 1.13 million metric tons, respectively [37]. Camels and the growing prevalence of lactose intolerance, in addition to the wellness trends and expanded distribution systems, make up the camel worldwide dairy market, worth 7.5 billion US dollars in 2023, which is growing due to heightened consumer awareness of the health benefits of CM [42,43]. In the modern day, camels have been found in regions where the environmental conditions have challenged the traditional pattern of livestock rearing [36]. This is where camels have been discovered to be an invaluable source of nutrition security alongside the high nutritional index, ease of digestion [44,45], in addition to other related benefits, which has made the interest of consumers to expand beyond the conventional pattern of use of the camel in the local and domestic market on a global level [46].

### 2.2. Unique Compositional Features Distinguishing Camel Milk from Bovine Milk

The unique compositional characteristics of CM that make it different from BM are multifaceted and result from basic differences in protein structure, lipid composition, carbohydrate content, and micronutrient profiles. Recent meta-analyses on the gross composition of CM, i.e., one-humped (dromedary) and two-humped (Bactrian) CM, have resulted in average compositional values of 3.17% protein, 3.47% fat, 4.28% lactose, 11.31% total solids, and 0.78% ash content [38]. At the protein level, CM has a fundamentally different casein (CN) profile than BM, with β-CN making up about 65% total CN content in CM compared to only 36% in BM, and α-CN making up 24% total CN content in CM compared to a greater proportion in BM [37,47]. The CN to whey ratio also shows a significant difference, with a ratio of 26:4:67:3 (*w*/*w*) for αs1:αs2:β:K-CN in CM compared to 38:10:36:12 (*w*/*w*) in BM [37]. Perhaps most significantly from an allergenicity standpoint, CM does not contain any β-lactoglobulin, the major allergenic protein in BM, and, hence, can be used by individuals with BM protein allergies [37,44,47,48]. The WP fraction of camel’s milk is an ingredient with bioactive compounds, α-lactalbumin (LA) (0.3 to 2.9 g/L, which comprises 50% of whey proteins (WP), serum proteins (35%) [37]. In addition to LF (0.18 to 2.48 mg/mL) [47,49], lysozyme (LZ) (228 to 500 µg/100 mL) [47], and IGs (1.64 to 2 mg/mL) [34,50] that together exhibit antimicrobial, immunological, and antiviral properties [37,47]. Notably, CM is found to have significantly greater content of LF and LZ than BM, which range between 0.080.5 mg/mL [47,51]. Additionally, camel’s milk is unique in containing peptidoglycan recognition protein (PGRP) at a concentration of 107 mg/L, which is not found in BM and is important in innate immunity [37,44,52]. The IG content of CM is of utmost importance; the IGG content of CM in early lactation is as high as 132 mg/L and 4.75 mg/L in mature milk [53], which is quite higher than the content of BM, which varies between 0.620.67 mg/mL [50].

From a lipid perspective, CM exhibits a number of advantageous characteristics that differentiate it from BM, most notably, the much smaller size of fat globules, which average 2.99 mm in diameter compared with the larger globules (2.8–4.6 mm) found in BM, but this is just one of the studies that have reported smaller sizes in CM [45,48]. The fat content of CM is around 3.47% [38], generally less than that of BM, which is 3–4%, and it is characterized by the higher cholesterol content of 30–37.1 mg/100 g, compared to that of higher levels in BM of 26–31 mg/100 g [37,47]. CM fat consists mainly of triglycerides (96%), phospholipids (1%), and cholesterol [47,53,54], with a good proportion of fat comprised of saturated and unsaturated lipids, a large percentage of long-chain fatty acids (92–99%) and unsaturated fatty acids (35–50%), with a minor amount of short-chain fatty acids [55]. Moreover, CM contains an extremely high level of polar lipids or phospholipids, such as phosphatidylserine, phosphatidylethanolamine, phosphatidylinositol, and sphingomyelin, content than other milk-producing animals, such as goat, sheep, and BM [47,55]. The homogenous composition of fatty acids and low carotene concentration, which allows CM to have a typical white color and a smooth texture, and their presence is favorable for the biological and immunological properties of CM, lowering inflammation and blood sugar level, in addition to decreasing the risk of cardiovascular diseases [56].

The carbohydrate profile of CM was also found to have some unique features, as the lactose content is 4.28% [38], which is relatively less than BM (4.7%) and significantly less than buffalo milk (5–6%) [37,48]. Besides lactose, CM is reported to contain a unique carbohydrate profile that consists of galactooligosaccharides, fucosylated oligosaccharides, galactose, fructose, and glucose [40]. This carbohydrate profile enhances the health benefits of CM and makes it an important subject of interest in food science and nutrition research [57]. The relatively low amount of lactose, in combination with the special composition of camel proteins, makes CM a convenient alternative for people with lactose intolerance, as they can consume CM without a significant problem [58]. Additionally, this may be because CM contains a lesser amount of casomorphins, and this subsequently leads to a slower intestinal motility process and affects the lactose digestion process [31,48,59].

CM has a favorable micronutrient profile, such as three to five times greater vitamin C than BM [40], higher iron (0.29–0.53 mg/100 g), zinc, and vitamin D3 (8 times higher than BM) [38,40,47], which are important for immunocompromised children with documented micronutrient deficiencies [21,22,23]. The compositional data are presented in Table 1.

## 3. Immunological Arsenal and Functional Advantages of Camel Milk

### 3.1. Heavy-Chain Immunoglobulins (IGG2, IGG3): Structural Uniqueness and Functional Superiority

IGs are antibodies composed of polypeptide chains that have a crucial role in the protection of the immune system from infections [67,67]. Literature revealed that IG content is about 2000 mg/L in CM, and the content is lower in buffalo, with about 460–1300 mg/L, and in bovine with about 100–800 mg/L [34]. Five classes of IGs are found in CM, including IGA, IGD, IGE, IGG, and IGM isotypes [68,69]. CM contains conventional IGG and non-conventional single-chain IGG (HCAbs) [70,71]. Structurally, the non-conventional single-chain IGG antibodies are significant for their high solubility and stability [72,73,74]. While conventional IGG antibodies consist of two identical heavy chains (Each chain includes: VH, CH1, CH2, and CH3) and two identical light chains (Each chain includes: VL and CL), the light chain (L-chains) and the first heavy-chain constant region CH1 are missing in the single-chain IGG antibodies [70]. HCAbs are bivalent and include variable domains that exhibit nanomolar binding affinities and undergo somatic hypermutation [75]. The HCAbs of IGG2 and IGG3 contribute about 75% of all serum IGG [70,76]. They are smaller than conventional antibodies, about one-tenth the size, and this reduced size enhances their ability to easily penetrate cell membranes, even those of bacteria [77]. The variable antigen-binding domains of IGG2 and IGG3 are known as (VHH) [78]. These IGs and their VHHs have advantages over common antibodies due to their biodistribution [79,80]. The masses of antigen-binding VHH domains are the smallest natural fragments known, with approximately 15 kDa [81]. VHH domains of HCAbs exhibit specific characteristics through their ability to substitute about three to four hydrophobic residues with more hydrophilic amino acids [82,83]. Interestingly, VHHs have a long complementary determining region (CDR) that penetrates deeply within enzymes and fully neutralizes it [84]. Of these complementary determining regions, the CDR3 are statistically longer than those found in conventional VH-VL antibodies [81].

### 3.2. Lactoferrin: Multifunctional Iron-Binding Glycoprotein

LF is a glycosylated protein that consists of about 700 amino acids [85] with a concentration-dependent thermal sensitivity where complete inactivation is observed at 85 °C, for 30 min [86]. Although partial retention is observed at milder conditions. Its multifunctional properties and processing stability are critical factors for therapeutic applications. It was known as “red protein from milk” [87]. Ferrous (Fe^2+^) or Ferric (Fe^3+^) are the metals that bind to LF, but it can also be found as free of Fe^3+^ (apo-LF) [88]. As a dietary supplement, LF is used in the field of infant nutrition and immunity [89]. Many factors, including genetic factors, environmental factors, and animal breeds, affect the content of LF in different milk sources [90]. LF content in CM is about 0.18 to 2.48 mg/mL [47,49], and the content is lower in BM with about 0.08–0.5 mg/mL [91,92], while the lowest content of LF is reported in donkey milk with about 0.08 mg/mL [93].

### 3.3. Synergistic Interactions Between Immunological Components

CM is functionally recognized for its crucial bioactivities, including bioactive peptides and key proteins such as LF, IGs, LZ, and lactoperoxidase, which all contribute to its ability to work as an antioxidant, antifungal, antiviral, antibacterial, and anti-inflammatory effects [34,94]. As CM antibodies comprise HCAbs, which have unique biophysical properties, they also provide practical advantages through different medical and biotechnological applications [70]. These antibodies are considered less immunogenic than many other mammalian antibodies [95], which means when they are used during the course of treatment in experimental models, they are less likely to enhance adverse reactions [96]. Pharmaceutically, it is recommended to use HCAbs of CM as substitutes for other animals’ therapeutic antibodies [97], in addition to their ability to be converted into a complete humanized antibody [98]. The single antigen-binding domains (VHHs) of these HCAbs may have a variety of applications in cancer diagnosis, treatment, and biosensor development [99,100]. As an efficient biomolecular agent, VHHs are used in diagnosis and therapy for the detection and treatment of bacterial, viral, and parasitic zoonosis through their ability to inhibit or neutralize these pathogens [72]. HCAbs are widely used for immunotherapy and the synthesis of promising vaccines [101]. These immunotherapy researchers focus on many immunity-related conditions, including many harmful viruses, inflammatory agents, and autoimmune diseases [88,102]. LF, derived from milk, has a variety of biological characteristics such as antioxidant, anti-inflammatory, antifungal, antibacterial, anti-parasitic, antiviral, and anti-cancerous properties [73]. Camel LF possesses strong antibacterial effects through its ability to sequester and chelate the iron ion, which is nutritionally needed for bacterial growth [103,104]. This mechanism prevents the proliferation of a wide range of bacteria, either Gram-positive or Gram-negative [105]. However, other actions besides iron holding include destabilizing the cell wall or blocking carbohydrate metabolism by a wide range of bacteria [106,107]. Literature revealed scientific evidence for the inhibitory effect of LF on the growth of *Listeria monocytogenes*, *Bacillus stearothermophilus*, *Bacillus subtilis*, *Pseudomonas aeruginosa*, *Shigella dysenteriae*, *Escherichia coli*, *Salmonella* spp., *Streptococcus aureus*, *Helicobacter pylori*, *Klebsiella pneumonia*, and *Clostridium* spp. [91,108]. LF demonstrates antiviral functions against many RNA and DNA viruses, through blocking virus cell receptors or through direct attachment to the virus surface. These targeted viruses include CMV, hepatitis B, C [109], coronaviruses including SARS-CoV-2 [110,111], human papillomavirus, rotavirus, human immunodeficiency virus (HIV), and HSV-1 [112]. As an antifungal, [113] explored that the LF of CM has an inhibitory effect against some infectious fungi. Significantly, the researchers of this study reported that camel LF effectively prevented fungal proliferation and growth by destroying their cell membranes. Moreover, LF has a key role in immunotherapy through modulating different immune cells’ maturation, including macrophages, lymphocytes, and neutrophils [114,115]. The bridging of innate and adaptive cell functions induces this modulation [88]. Moreover, camel LF demonstrates an anti-arthritic role against interleukin-1 (IL-1) and kappa B signaling events and nuclear factors, which enhance the activity of cartilage gaps in human osteoarthritis [116]. CM LF was also investigated to suppress prostaglandin E2 synthesis and cyclooxygenase-2 expression of activated osteoarthritis chondrocytes in humans [116]. LF has anticancer and anti-arthritic activities [116,117,118], and CM exhibited therapeutic potential in the management of diabetes [119,120,121,122]. However, application to immunocompromised pediatric populations has yet to be investigated, which is a critical translational gap that is addressed in Section 5.

### 3.4. Bioavailability and Digestibility Advantages in Compromised Digestive Systems

The bioavailability and digestibility benefits of CM in compromised digestive systems are important functional characteristics that make CM a potentially useful nutritional intervention in susceptible pediatric populations, especially those with immature or impaired gastrointestinal function [44,123]. The superior digestibility of CM is attributed to several synergistic factors, such as the composition of the protein, the structure of the lipids, and the presence of bioactive protective proteins. The high β-CN content of CM that dominates the CN fraction at 65% of total CN is particularly advantageous since β-CN is subjected to more efficient peptic hydrolysis in the gastrointestinal tract compared to the other CN fractions, thus proving itself to be potentially healthier and less allergenic to human consumption [47,123]. Studies using in vitro infant gastrointestinal digestion systems have shown that CM proteins are equally digestible, similar to bovine and human milk proteins, with a low degree of gastric proteolysis observed and the formation of a single soft clot in CM compared to the formation of the harder and more fragmented curds that are typical of BM digestion [44,51]. This softer curd formation, which is due to the high β-/αs-CN ratio and unique CN micelle structure in CM (which are about double in size than the bovine CN micelles, ranging from 260,300 nm versus 100–140 nm), allows for more convenient gastric emptying and more efficient access by enzymes during intestinal digestion [37,44]. The remaining soluble milk proteins in the gastric digesta are subjected to fast and extensive digestion in the intestinal phase, while the proteins in the CM clot undergo slow hydrolysis and consequently attain a sustained release of amino acids [44,51]. The absence of β-lactoglobulin protein in CM may be very important from the allergenicity and digestibility points of view [48,51], because this protein is the major allergic agent in BM, and its absence reduces the risk of allergic reactions and increases gastrointestinal compatibility, especially in children allergic to BM protein [44,51,123,124].

The superior digestibility of fat in CM is mainly attributed to the markedly smaller size of the fat globules, and this facilitates the increased surface to volume ratio and the greater accessibility of pancreatic and gastric lipase, resulting in the rapid release of free fatty acids [45,125,126]. It has been compared with other milk types and studies. CM fat is more digestible compared to bovine and buffalo milk, which is especially important for populations of infants who have an immature digestive system and produce lower concentrations of pancreatic lipase [45]. The large globule size of BM fat (2.8–4.6 µm) requires more effort from the infant’s digestive system, and during the digestion stage [124]. Moreover, fat globules of BM tend to destabilize and aggregate into larger particles, causing more resistance to the activity of digestive enzymes [124]. While the small and homogenous fat globules in CM remain available to the digestive enzymes throughout the digestive process [48]. Furthermore, the high pH values in the stomach of infants and the lower concentration of pancreatic lipase, which is responsible for the delay in the process of BM fat digestion, have less effect on the digestion of CM fat as a result of its superior structural characteristics [48].

The protective proteins found in CM’s whey fraction offer a great deal to the digestibility and bioavailability benefits of CM in impaired digestive systems by having multiple layers of gastrointestinal support. LF, found in concentrations of 0.18–2.48 mg/mL in CM (considerably higher than the levels found in BM), has antimicrobial, anti-inflammatory, and immunomodulatory effects [47,49,55,127]. This supports intestinal health and the defense against pathogenic bacteria, even at the low pH values that are measured in the stomach and intestines, as a result of its capacity to scavenge iron and its capacity to deplete free iron that can be utilized in support of bacterial growth [47,127,128]. LZ, in a concentration of 228–500 µg/100 mL (imaginably higher than about 11 µg/100 mL in BM) [61,62] acts by hydrolysis of the β(1-4)-glycosides present in peptidoglycan by cleaving the bond between N-acetyl-D-glucosamine and N-acetylmuramic acid, ultimately leading to inhibition of bacterial growth and forming part of the natural antimicrobial protection of the gastrointestinal tract [47,51,129]. The IGs present in CM, especially IGG, IGA, and IGM, provide passive immunity and also aid in the digestive system by destroying the antigens, which are subsequently overwhelmed and digested by macrophages, protecting against various infectious diseases [47,50,73]. The unique existence of PGRP in CM but not BM provided an extra layer of innate immunity against the infectious agent through its action on gram-positive bacteria and other microorganisms [44,52].

The tolerance of CM in lactose-intolerant individuals, despite the relative quantities of lactose (4.18–4.46%), is another important digestibility benefit that is especially applicable in pediatric groups with digestive sensitivities. Clinical observations have shown that people who develop symptoms on ingestion of BM can use CM without any undesirable reactions, with the tolerance possibly due to the distinct protein composition of CM, which leads to reduced generation of casomorphins that influence intestinal motility and the kinetics of lactose digestion [31,48,59]. In a clinical study on 25 patients who are intolerant to lactose, CM was successfully consumed without any adverse symptoms, which indicates that it can be taken into consideration by individuals who are lactose intolerant [130]. The improved digestibility in CM is further backed by studies that the proteins and fats of CM are more available to the digestive enzymes, thus facilitating faster gastric emptying and efficient digestion [48]. Comparative studies that investigate the protein digestibility under simulated gastric conditions of infants have confirmed that CM and also goat milk form softer curds than BM for the ease of more efficient protein hydrolysis and nutrient absorption in babies with immature digestive systems [44,48]. The totality of evidence from compositional analyses, *in vitro* digestion studies, and clinical observations has collectively shown that CM has inherent structural and compositional characteristics that give it superior digestibility and bioavailability in compromised digestive systems to make it a potential functional food for immunocompromised pediatric populations that not only require optimal nutrition but also need enhanced tolerance to dietary proteins. However, these advantages of digestibility are processing-dependent. Thermal treatments in excess of 85 °C result in complete denaturation of LF and protective proteins [86,131]. This could have repercussions for the gastrointestinal tolerance mechanisms that make CM suitable for compromised digestive systems. Section 4 focuses on the modification of these functional properties as a result of processing.

## 4. The Processing Dilemma

### 4.1. Industrial Necessity of Milk Processing for Safety and Shelf-Life

The industrial need for thermal processing, however, is in direct conflict with preservation of bioactive proteins that form the basis of CM’s therapeutic application for immunocompromised pediatric populations, and presents a fundamental dilemma in which microbiological safety and preservation of bioactivity exist in inverse relationship [132,133,134]. Thermal processing of CM may increase the digestibility of CM by denaturing some of the proteins present in milk [135]. While at the same time degrading bioactive constituents, such as IGs, LF, and some enzymes, and the extent by which these are degraded is directly proportional to the intensity and duration of the heat treatment [133,134]. CM IGs, especially IGG, which is the most abundant and therapeutically relevant class of IGs present in 132 mg/L in early lactation and 4.75 mg/L in mature milk [136]. These show differential thermal stability compared to BM IGs, in which CM IGG lost 68.7% of its activity after pasteurization at 75 °C for 30 min, while bovine and buffalo milk IGG completely lost all activity under the same conditions [86]. When CM is heated to 100 °C for 30 min, it results in a complete loss of the activity of antimicrobial factors, such as IGs, LF, and LZ, rendering the milk microbiologically safe but having no therapeutic potential [86,131]. Apart from safety, thermal processing also helps to increase CM shelf-life from 2–3 days for raw milk at 4 °C to 10–14 days for pasteurized (72 °C/15 s) and several months for UHT-treated products [133]. However, this shelf-life extension happens simultaneously with the degradation of bioactive protein, further exacerbating the processing paradox for immunocompromised populations that need stability of storage over long periods of time and therapeutic efficacy. The Processing technologies for camel milk are shown in Table 2.

LF, which is found in a significantly higher concentration than found in BM, is another important bioactive protein that is very susceptible to thermal processing because its antimicrobial activity is attributed to direct binding to microbial membranes [47]. LF chelates to iron, which is essential for bacterial growth, and inhibits the ability of microorganisms to adhere to epithelial cells [103]. Milk heated at 85 °C for 30 min induces total inactivation of LF in camel, bovine, and buffalo milk [86], whereas the remaining activity of LF in human milk is 91% and 22%, respectively, when heated at 57 °C for 30 min, and 62.5 °C for 30 min [137]. Furthermore, heat treatment of CM at 63 °C and 98 °C for 60 min totally causes the denaturation of protective proteins such as LF, lactoperoxidase, and PGRP, whereas this effect is negligible after 63 °C for 60 min heating treatments [131], demonstrating a critical temperature below which bioactivity is irreversibly lost [138]. The thermal sensitivity of CM WP follows a hierarchy pattern with α-LA being the most heat-sensitive protein among the other proteins, followed by PGRP and SA [138], such that the concentration of α-lactalbumin and SA decreases after heating at 90 °C for 120 min [138,139]. LZ, which is found in CM at concentrations of 228–500 μg/100 mL, is significantly higher than that found in BM of 11 μg/100 mL [86]. These concentrations were found to loss 56% of their activity in CM, 74% in BM, and 81.7% in buffalo milk when subjected to heat treatment at 85 °C for 30 min, demonstrating greater heat-resistance of antimicrobial factors in CM compared to those in bovine and buffalo milk [86].

### 4.2. Vulnerability of Bioactive Proteins to Processing Conditions

The vulnerability of CM bioactive proteins to processing extends beyond simple thermal denaturation to encompass complex structural modifications, including protein aggregation, disulfide bond formation, and conformational changes that fundamentally alter biological function [132]. Moreover, the lack of β-lactoglobulin and lower concentration of κ-CN in CM resulted in different responses to heat treatment compared to BM at both laboratory and industrial scales [132,140]. A combination of the heating treatment and acidification of camel whey leads to a sudden disappearance of α-LA and appearance of a number of intermediate proteins, such as dimers and trimers of α-LA proteins [64,141]. In addition, the associated protein released during heating treatment and prior to aggregation, and acid whey has higher denaturation in comparison to sweet whey, regardless of heating temperature [64,142]. This confirms that acid whey is defined by the greater thermal sensitivity than the sweet whey, since the thermal sensitivity of camel WPs is higher than that of the bovine WPs, particularly at neutral conditions [143]. All of this contributes to the formation of the large aggregates: the open structure of the camel α-LA molecule, the lower electrostatic repulsion of this protein, which is close to its isoelectric point [141,144]. Since purified camel α-LA isolated from CM becomes more flexible when subjected to acidic conditions, irrespective of the heating temperature, due to its negative charge decreasing, and it is in a molten globular state at low pH values [64,141,144]. Recent investigations have found several structural variations between camel and bovine α-LA depending on different denaturing conditions in food processing, including pH, heating temperature, and guanidine hydrochloride-mediated denaturation [141]. These conditions, where camel α-LA showed higher stability towards thermal treatments and pH-mediated denaturation, but were less stable towards guanidine-mediated denaturation, showing faster aggregation and a more disordered structure when compared to its bovine counterpart [64,141,145]. The heat treatment of camel caseinates results in degradation and denaturation of individual CNs, including α-CN and β-CN, at temperatures of 90 °C and 100 °C for 30 min [146]. This treatment is likely linked to the liberation of resulting peptides, whereas β-CN and α-LA are the main proteins in colloidal and soluble fractions of CM, respectively [64]. Moreover, CM proteins behave differently compared to bovine proteins, whereas heating treatment of milk significantly affects α-LA, then on PGRP, and serum albumin, with a relative thermal sensitivity of WPs as compared to CNs [64,143].

Non-thermal processing technologies have emerged as potential alternatives to traditional heat treatments for preserving bioactive components while ensuring microbiological safety [147,148]. Though each presents its own advantages and limitations for therapeutic applications in immunocompromised pediatric populations [132,133,148]. High hydrostatic pressure processing (HPP), which involves subjection of milk to pressures ranging from 200,800 MPa at processing temperatures of 0–40 °C for exposure time of a few seconds to 20 min, can inactivate both pathogenic and spoilage microorganisms to obtain microbiological quality similar to pasteurized milk (72 °C for 15 s) [149]. For pressure between 400,600 MPa with a shelf life of thermally pasteurized milk of 10 days at 10 °C, being obtainable using high-pressure treatment of at least 400 MPa that reaches CM above 200 MPa for 5 min at 40 °C, which reduces microbial contamination without changing color, aroma, or taste [150]. Although CM is clotted at high pressure up to 300 MPa, which lowers the effect of pressure on bacterial lethality [149,150]. Critically for bioactive protein preservation, high pressure (200–800 MPa), does not cause any changes in the composition of CM [149]. Furthermore, 200 MPa high-pressure treatment leads to a lower degree of denaturation of camel WP when compared with UHT treatment, while 400 to 800 MPa pressure leads to a significant increase in denaturation of α-LA up to 32.5%, which is still considerably less than the UHT-treated CM [149]. While SA and LF are more pressure resistant and have low denaturation of 3.94% and 2.93%, respectively [149,150]. The stability of camel WPs is greater than that of BM when they are subjected to high-pressure conditions. With high-pressure treatment at pressures greater than 200 MPa and at 0–40 °C, there is little deviation from the covalent bonds [149]. However, a change in hydrophobic and ionic interactions, which leads to maintaining the tertiary and quaternary structures of molecules, although molecules having α-helices structure are less stable to pressure as compared to β-sheets, and denaturation of WPs can be reversible at pressures up to 101–304 MPa and irreversible above 304 MPa [132,151,152].

Ultrasonication is an alternative non-thermal technique that inhibits bacterial growth through acoustic cavitation while causing a reduction in fat globule size and enhancing antioxidant capacity of milk [153,154,155]. In addition, ultrasonication demonstrates efficacy against some microorganisms but has a relatively low efficacy against microorganisms that produce spores and those that survive high temperatures relative to thermal treatment and, thus, results in moderate shelf-life extension [155,156,157]. High-intensity sonication leads to structural change in proteins in addition to destabilization of LF and LZ, thereby reducing nutritional and functional characteristics of CM [154,155]. Moreover, the development of acoustic cavitation also generates free radicals that enhance lipid peroxidation, leading to the undesirable flavor, aroma, and color changes [155,157]. Sensitive vitamins such as vitamin C and some B complex vitamins are susceptible to degradation during prolonged sonication, and from the practical point of view, ultrasonication is costly, energy-consuming, and difficult to scale up for large-scale processing with non-uniform treatment resulting in inconsistent microbial inactivation [132,155,157]. The UV-C irradiation with wavelengths of 200–280 nm has a higher killing potential to different microorganisms [158]. Application of UV-C leads to inactivation of pathogens that cause spoilage of CM by affecting DNA lesions or disruption of cytoplasmic membrane integrity and cellular enzyme activity inhibition [155,158]. Though UV-C application as a non-thermal treatment to CM can be affected by its limited capability to pierce turbid fluids [159]. Studies evaluating UV-C treatment on CM composition and pathogenic bacteria showed that *Salmonella typhimurium* and *E. coli* O157:H7 showed the same level of resistance of 3.9 log reductions at 12.45 mJ/cm^2^ UV-C intensity, with limited effect on conjugated linoleic acid and formation of volatile compounds [160]. Gamma irradiation of CM whey powder at a dosage of 9 kGy reduced microbial load and improved shelf life [161]. Although irradiation is effective in the destruction of pathogenic bacteria, viruses, and parasites, and the retention of more nutrients when compared with thermal treatments [162]. Thermal treatments can cause minor alterations in taste, odor, and color that may affect consumer acceptability, and public concern and misconceptions about radiation safety may limit its acceptance and use [133,147].

**Table 2:** Processing technologies for camel milk: Features, advantages, and limitations.

Technology	Conditions	Microbial Reduction	Bioactive Retention	Advantages	Limitations	References
Thermal						
LTLT pasteurization	63 °C/30 min; 65 °C/30 min	Destroy non-spore-forming pathogens; shelf life 2–3 weeks (7–20 days) at 4 °C	65 °C/30 min: no significant effect on LF and LZ, but IGG significantly loss 68.7% of activity	Simple; better retention vs. higher-heat; feasible for small-scale	Requires cold chain; spores survive; IGG is still reduced	[86,155,158,163]
HTST pasteurization	72 °C/15 s	Kill of non-spore formers; shelf life 2–3 weeks (>10–15 days at 4 °C)	Limited LF denaturation with HTST (1.13%)	Industrial standard; good quality/safety balance	Insufficient to destroy spores; moderate bioactive protein loss; affects heat-sensitive vitamins (C and B-group)	[133,149,155,163,164]
High-temperature holding	85–90 °C/15–30 min	Strong microbial reduction	85 °C/30 min: complete loss of LF and LZ loses 56% activity	Improved viscosity in fermented products	Significant bioactive protein loss	[86,149]
UHT/sterilization	138–145 °C/1–10 s	Destroys all non-spore-forming bacteria, most spores, and spoilage enzymes; shelf life 6–9 months under cold storage	At 138 °C/4 s: destroys majority of IGs; complete denaturation of protective proteins (LF, lactoperoxidase, PGRP); milk fails to coagulate with rennet	Extended shelf life without refrigeration; sterilization-level safety	Significant bioactive protein degradation; development of off-flavors (volatile sulfur compound; protein sedimentation; poor coagulation	[131,133,158,163]
In-Container Sterilization	115–120 °C/10–30 min	Destroys all spore-forming and non-spore-forming bacteria; inactivates spoilage enzymes	100 °C for 30 min: total loss of antimicrobial factors activity; near-complete loss of heat-sensitive bioactive compounds	Complete sterilization; extended shelf life	More severe processing; maximum bioactive protein loss; highest energy consumption; complete loss of therapeutic properties	[86,158]
Non-thermal						
UV-C	12.45 mJ/cm^2^; 200–280 nm	At 12.45 mJ/cm^2^: both *E. coli* O157:H7 and *S. typhimurium* show 3.9-log reduction	Minimal impact on major components; limited effect on CLA and formation of 3 new volatile compounds (p-Cresol, Octanoic acid, Tetradecanal)	Non-thermal process; preserves more nutrients than pasteurization; no chemical residues	Limited penetration in turbid milk; dose may be insufficient for regulatory 5-log targets	[158,160,165]
Ultrasonication	900 W, 20 kHz, 100% power level, 15 min	Complete inactivation of *E. coli* O157:H7 (6-log reduction); 4.4-log reduction for *S. typhimurium*; 2-log reduction in total aerobic bacteria	Destabilization of LF and LZ	Easy and low cost; no adverse effects on nutritional potential; improves bioactive peptide formation	Low efficacy against spore-forming microbes; moderate shelf-life extension only; protein destabilization; expensive and energy-intensive	[154,157,158,160]
Gamma Irradiation	9 kGy	Effective reduction of bacterial contamination	Total camel IGG reduced by 13%; no effect on major whey proteins	Effective pathogen inactivation; improved milk safety; non-thermal preservation	Sensory changes possible; regulatory restrictions; public acceptance issues; equipment costs	[133,158,161]
High-pressure processing (HPP)	400–600 MPa, 20–30 °C/15 min	Equivalent to pasteurization (72 °C/15 s)	α-LA: 32.5% denaturation; LF denaturation remains low (2.93%); better retention than thermal processing	Minimal thermal damage; can improve some functional properties	High capital cost; batch processing; can affect proteins at high pressure	[149,150]
Combined						
Thermosonication	41 °C/5.9 min at 81 W	Enhanced microbial inactivation compared to ultrasonication alone	Moderate retention vs. thermal alone	Synergistic antimicrobial effect; improved rheological properties; better milk stability than ultrasonication alone; lower temperature than conventional thermal processing	Needs optimization of temperature-sonication parameter; still limited against spores; equipment complexity; energy consumption	[158,166]

### 4.3. Current Knowledge Gaps in Structure-Function Relationships Post-Processing

Current knowledge gaps in the structure-function relationships of CM bioactive proteins post-processing represent a critical barrier to developing evidence-based therapeutic applications for immunocompromised pediatric populations [132,133]. The existing research presents contradictory findings, focuses predominantly on compositional changes rather than functional outcomes, and lacks systematic investigation of the relationships between processing-induced molecular modifications and therapeutic efficacy in vulnerable populations. The scientific literature discloses some of the basic disagreements on the heat stability of CM proteins, where some scientists reported that CM WPs are more sensitive to heat treatment, with denaturation rates faster than those of BM [131,149,167]. Whereas other scientific investigations have demonstrated that camel WPs are much more heat resistant than those in BM after pasteurization at different temperatures (65, 75, 85, and 100 °C for 10, 20, and 30 min [86,168]. Other researchers have noted that during pasteurization at 60, 70, 80, 90, and 100 °C, no significant difference in the heat stability of liquid whey separated after CM versus BM was found [143]. Whilst preliminary studies of dried whey show that camel WPs are slightly more prone to heat denaturation than bovine whey, with factors including stage of lactation, camel breeds, feeding conditions, and geographical location potentially responsible for the conflicting results [149,169]. The effect of heat treatment on IG activity has been shown to vary substantially, with Elagamy [86] reporting that pasteurization at 65 °C for 30 min has no significant effect on LF and LZ in camel, bovine, and buffalo milk, while IGs were significantly affected. Whereas Levieux, Levieux [170] and Omar, Harbourne [149] found that WPs in early CM (first week of lactation) were more heat-sensitive than those in milk of camels after three months of lactation, and this result was explained by the high concentration of IGG in early milk (12.6 mg/mL) relative to later lactation milk (0.5 mg/mL) of camels. These contradictory results highlight the great need for standardization of methodologies and systematic studies that take into account the multiple variables that influence protein stability, such as lactation stage, breed differences, geographical differences, and processing conditions [169,170].

The gap between molecular changes (i.e., processing-induced) and biological functionality (i.e., bioactivity) is perhaps the most important knowledge gap as the body of research has mainly focused on measuring the denaturation of protein through techniques like differential scanning calorimetry, electrophoresis, and high-performance liquid chromatography and is rarely correlated with the retention of specific biological activities relevant for therapeutic application in immunocompromised populations. While studies have reported that the results of two-dimensional differential gel electrophoresis mass spectrometry of CM indicate a high level of similarity of isoelectric point, molecular weight and protein fluorescence intensity of raw milk, milk heated at 65 °C for 1 h and milk heated at 98 °C for 1 h, respectively, studies have reported it to be more similar with 2-dimensional differential gel electrophoresis mass spectrometry of CM [131]. The functional implications of these molecular changes for antimicrobial activity, immunomodulation, anti-inflammatory properties, and therapeutic efficacy in immunocompromised pediatric patients remain largely unexplored. Thermal processing decreases bioactive components and therapeutic properties; however, dose responses between heat damage and functional retention have not been established [133,155]. The potential for bioactive peptides generated during gastrointestinal digestion of processed CM to compensate for processing-induced losses of native protein bioactivity represents an unexplored area. As in vitro digestion studies have identified numerous bioactive peptides [33,34] with ACE-inhibitory, antimicrobial, antioxidant, DPP-IV-inhibitory, and immunomodulatory functions in processed CM, but whether processing conditions affect the generation and bioavailability of these peptides in vulnerable pediatric populations remains unknown [44,171].

The lack of clinical evidence through data on the association of processing choices with therapeutic outcomes among immunocompromised pediatric populations is a critical knowledge gap that needs to be addressed to foster evidence-based processing strategies specific to therapeutic applications. While multiple studies have documented the therapeutic effects of raw or minimally processed CM in pediatric populations. This includes improvements in autistic children’s glutathione, superoxide dismutase, and myeloperoxidase levels following CM consumption, and improvements in tuberculosis patients [32,172]. This is evidenced by negative bacteriological findings, improved radiological reflections, and increased micronutrients and IG levels. The specific processing conditions employed in these studies are rarely reported with sufficient detail to enable replication or optimization [31,120]. The biological activities of fermented CM have been shown to be preserved during storage for 21 days at 4 °C [173,174]. Moreover, protein hydrolysates from CM demonstrate higher antioxidant, antimicrobial, and hypertensive activities compared to intact protein sources when hydrolyzed by pepsin, chymotrypsin, papain, bromelain, and alcalase [175,176]. This suggests that certain processing approaches may actually enhance rather than diminish bioactivity, yet systematic investigations correlating processing parameters with therapeutic outcomes in immunocompromised children are absent from the literature [147,177]. The development of optimized processing strategies requires understanding not only which bioactive proteins must be preserved and to what extent, but also which molecular modifications are acceptable or even beneficial for therapeutic efficacy in specific immunodeficiency conditions affecting pediatric populations, yet this fundamental structure-function information linking processing-induced changes to clinical outcomes in vulnerable children remains critically underdeveloped.

**Figure 2:**
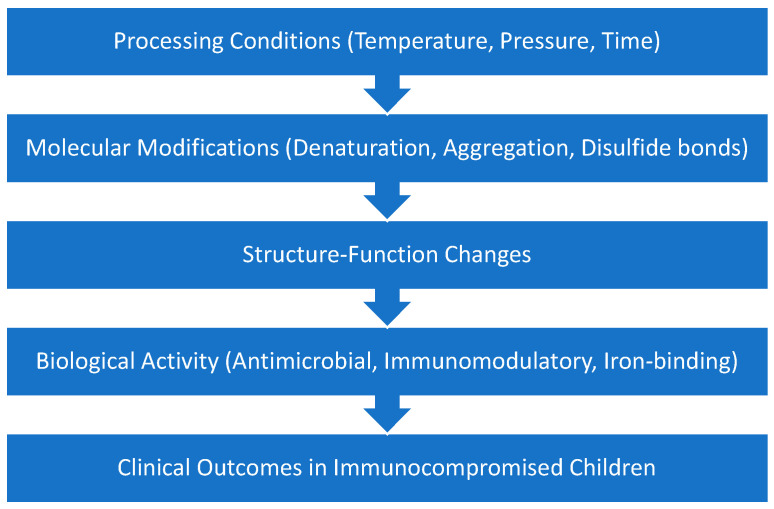
Conceptual Framework.

The lack of standardized post-processing evaluation protocols creates methodological inconsistencies that hinder comparison across studies and the development of unified processing recommendations [132]. Future research should systematically explore the processing approaches for therapeutic CM, addressing the optimization of physicochemical and functional properties without diminishing bioactive components. Furthermore, the determination of shelf life for immunocompromised populations, as well as the exploration of combined thermal, non-thermal, and biotechnological approaches that provide a balance between safety and bioactivity, would be investigated [147,178]. In addition, in-depth investigation of structure-function relationships between processing-induced molecular modifications and maintenance of clinically relevant therapeutic activities, especially in immunocompromised pediatric populations, is essential in order to optimize processing strategies that ensure both microbiological safety and clinical efficacy. The conceptual framework. Is shown in Figure 2.

### 4.4. Summary Key Research Gaps

(1) Incongruous thermal stability data because of non-standardized methodologies [86,143,149,168].
(2) Lack of functional bioactivity assessment post-processing because most studies measure denaturation, not therapeutic efficacy.
(3) Unknown effects of processing on bioactive peptides generation during digestion [171].
(4) No clinical trials between processing parameters and treatment outcome in immunocompromised children.
(5) Standardized safety markers for CM pasteurization verification not available [163,179].

### 4.5. Future Research Directions: Processing Optimization Strategies

Priority research areas are: (1) standard evaluation protocols correlating processing-induced molecular alterations and retention of antimicrobial and immunomodulatory activities [132,147]; (2) clinical evaluation in immunocompromised pediatric populations of raw vs. optimal processing & efficacy of CM in terms of safety and therapeutic efficacy; (3) investigation of concerted technologies (HPP + fermentation) that may increase rather than reduce bioactivity [171,173,180]; (4) development and validation of the use of CM-specific pasteurization marker for replacement of inadequate alkaline phosphatase testing [163,179]; (5) elucidation of processing effects on bioactive peptide generation during gastrointestinal digestion in vulnerable populations [171].

## 5. Study Rationale

### 5.1. Critical Need for Understanding of Processing-Induced Modifications

In spite of an increasing scientific understanding of the therapeutic value of CM, an important knowledge gap exists between food processing science and clinical use in immunocompromised pediatric populations. CM contains unique immunological components, including heavy-chain-only antibodies (IGG2, IGG3) up to 75% of total serum IGs [68] with exceptional immunological stability and unique epitope recognition capabilities due to the single domain VHH structure [70,181,182]. However, the fate of these bioactive immunoproteins during industrial processing is not sufficiently characterized, which represents a real limitation to their therapeutic application [32,37]. The issue at the basis of this research is the processing paradox: the thermal treatments necessary for microbiological safety, which are a non-negotiable value in immunocompromised children who have a 20-fold greater susceptibility to salmonellosis compared to healthy individuals [183], can also affect the functionality of immunoproteins [21]. CM IGG has 68.7% of its activity remaining after pasteurization at 75 °C for 30 min, while bovine and buffalo milk IGG have completely lost all activity under the same conditions, showing better but incomplete thermal stability [86]. Pasteurization at 72 °C for 15 s only leads to 10–30% loss of activity of IGs in BM [184], whereas UHT treatment at 138 °C for 4 s causes the elimination of the majority [185,186]. LF is even more sensitive: heating at 85 °C for 30 min results in complete inactivation in CM, while during heating at 63 °C and 98 °C for 1 h, protective proteins such as LF and lactoperoxidase are completely denatured [131,187]. Most of the current processing guidelines developed mostly for BM do not adequately address the unique structural characteristics of CM, especially poor heat stability with a heat coagulation time of only 2–3 min at 120–130 °C [188]. A critical challenge is the inadequacy of the conventional method of verification of pasteurization. Alkaline phosphatase, which is the standard marker of BM pasteurization, is still active following thermal treatment in CM, making it unsuitable for safety verification for CM pasteurization [163]. Alternative enzymes such as g-glutamyl transferase and lactoperoxidase have been suggested, but standardized testing protocols that are applicable for clinical use are still in development [57,179].

The absence of CM-specific safety standards and the lack of validated markers for pasteurization [163,179], in addition to the lack of controlled clinical trials in immunocompromised children [21,27] currently limit clinical adoption. Establishment of evidence-based processing protocols requires: (1) international harmonization of safety testing methods; (2) pediatric clinical trials of processing-defined CM products; (3) regulatory frameworks for functional food claims specific to immune-deficient populations.

### 5.2. Optimization Opportunities for Therapy Applications

Emerging non-thermal technologies such as HPP, pulsed electric fields (PEF), and ultrasonication are very promising alternatives. HPP (400–600 MPa) is used to achieve a microbiological quality similar to that of pasteurized milk (72 °C for 15 s) with a limited level of protein denaturation [37,151,163]. Treatment at 600 MPa for 15 min at 20–30 °C kills most of the vegetative cells without high-temperature protein destruction [149,150]. Pulsed electric field treatment has a 75–100% retention of bioactive proteins with little perturbation in the structure [189]. Recently, evidence shows that fermented CM from milk heated at 90 °C showed the greatest anti-bacterial activity against *S. Typhimurium* and improved antioxidants, suggesting strategic combinations of thermal-fermentation may preserve or increase bioactive properties [180]. However, systematic investigation of these technologies specific to CM immunoproteins is limited, with no studies directly comparing processing-induced modifications with therapeutic outcomes in immunocompromised pediatric populations. This addresses the urgent clinical need for primary immunodeficiency diseases, which affect approximately 2.8 per 1000 hospitalized children in the United States, with 17,234 hospitalized children affected by primary immunodeficiency diseases in the US, by means of 4.85% in-hospital mortality and diagnostic delay contribute to 28.4% mortality in confirmed cases in resource-limited settings [2,3]. HIV-infected children show cumulative prevalence of underweight (41.6%), wasting (24.65%), and stunting (49.68%), and children with cancer have malnutrition rates ranging from 6% to 51%, with overall poorer survival [14,18]. Children with SCID have failure to thrive in 54–88% of cases, and resting energy expenditure is common (54%) and exceeds 150% of predicted values (33–67%) [21]. These vulnerable children need safe and effective nutritional interventions, but current evidence on immune-nutrition is only available in preterm neonates without primary immunodeficiency, perinatally acquired HIV, or active malignancy [21,27].

## 6. Conclusions and Future Perspectives

CM contains unique immunoproteins (heavy-chain IGs for 75% of total IGs, LF at 3–5 times the BM concentrations) with demonstrated therapeutic potential. Thermal processing helps to ensure microbiological safety, but degrades the bioactive proteins in a way that is proportional to the temperature and time. Structure-function relationships of processing-induced molecular modifications to retention of clinically relevant bioactivities are poorly characterized. No clinical trials have examined processed CM in immunocompromised pediatric populations, and standardized processing protocols that balance safety with therapeutic efficacy do not exist. A number of developments need to be conducted, including: (1) Systematic investigation of non-thermal technologies (HPP, PEF) with functional bioactivity assessment; (2) controlled pediatric trials comparing processing methods; (3) CM specific pasteurization validation markers; (4) evidence-based processing optimization for therapeutic applications; (5) regulatory frameworks that can enable clinical translation in camel-producing regions where these vulnerable populations reside.

## Figures and Tables

**Table 1:** Compositional comparison of camel and bovine milk with relevance to immunocompromised pediatric populations.

Component	Camel Milk	Bovine Milk	Functional Relevance for Immunocompromised Children
IGG (mg/mL)	4.75 [53]	0.62–0.67 [60]	Passive immunity, pathogen neutralization
LF (mg/mL)	0.18–2.48 [47,49]	0.08–0.5 [47,51]	Iron sequestration, antimicrobial, immunomodulation
LZ (µg/100 mL)	228–500 [47]	11 [61,62]	Antibacterial, mucosal defense
β-lactoglobulin	Absent [37,48]	4.4 [63,64] (major allergen)	Reduced allergenicity, improved tolerance
PGRP (mg/L)	107 [37,52]	Absent [37]	Innate immunity against Gram-positive bacteria
Vitamin C (mg/100 g)	4.80–5.95 [38]	2 [65]	Antioxidant, immune support
Iron (mg/100 g)	0.29–0.53 [38]	0.045 [66]	Anemia prevention in malnourished children

## Data Availability

The original contributions presented in this study are included in the article. Further inquiries can be directed to the corresponding author.

## Abbreviations

The following abbreviations are used in this manuscript:

CM	Camel milk
BM	Bovine milk
IG	Immunoglobulin
LF	Lactoferrin
PGRP	peptidoglycan recognition protein
SCID	Severe combined immunodeficiency
IEI	Inborn errors of immunity
WHO	World Health Organization
SA	Serum albumin
LA	Lactalbumin
CN	Casein
WP	Whey proteins
LZ	Lysozyme
